# ChIPseqR: analysis of ChIP-seq experiments

**DOI:** 10.1186/1471-2105-12-39

**Published:** 2011-01-31

**Authors:** Peter Humburg, Chris A Helliwell, David Bulger, Glenn Stone

**Affiliations:** 1 Department of Statistics, Macquarie University, North Ryde, NSW 2109, Australia; 2 CSIRO Mathematical and Information Sciences, North Ryde, NSW 2113, Australia; 3 CSIRO Plant Industry, GPO Box 1600, Canberra 2601, Australia; 4 The Wellcome Trust Centre for Human Genetics, Oxford OX3 7BN, UK

## Abstract

**Background:**

The use of high-throughput sequencing in combination with chromatin immunoprecipitation (ChIP-seq) has enabled the study of genome-wide protein binding at high resolution. While the amount of data generated from such experiments is steadily increasing, the methods available for their analysis remain limited. Although several algorithms for the analysis of ChIP-seq data have been published they focus almost exclusively on transcription factor studies and are usually not well suited for the analysis of other types of experiments.

**Results:**

Here we present ChIPseqR, an algorithm for the analysis of nucleosome positioning and histone modification ChIP-seq experiments. The performance of this novel method is studied on short read sequencing data of *Arabidopsis thaliana *mononucleosomes as well as on simulated data.

**Conclusions:**

ChIPseqR is shown to improve sensitivity and spatial resolution over existing methods while maintaining high specificity. Further analysis of predicted nucleosomes reveals characteristic patterns in nucleosome sequences and placement.

## Background

**Figure 1:**
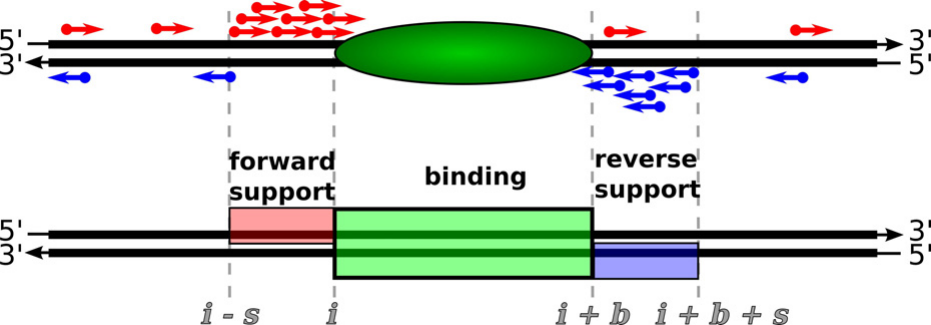
**Nucleosome model**. Schematic representation of a nucleosome (top) and corresponding binding site model (bottom). The signature of a protein binding event with high read density upstream and downstream of the binding site is partitioned into three regions. The support regions on the forward and reverse strand are flanking the binding region, capturing the peak in read density on the respective strand.

The recent advent of high-throughput sequencing technologies has enabled genome-wide studies of DNA-binding proteins at high resolution. In such studies the protein of interest is isolated together with a fragment of bound DNA, which is then separated from the protein and sequenced. This approach has been used to investigate several different proteins including the positioning of nucleosomes [1-4]. For this type of experiment DNA is typically digested with micrococcal nuclease (MNase) before isolating nucleosome-sized DNA fragments (~150 bp) that are then sequenced. This is the application considered here. Each nucleosome is expected to produce several sequence reads of approximately 35 - 100 bp from both strands. This leads to peaks in read density on either side of the nucleosome with the extent of the peaks and the distance between the two peaks determined by the length of DNA fragments and binding site. Since the DNA fragments produced by an MNase digest of nucleosomes are selected to be similar in length to the actual binding site the resulting peaks in read counts are expected to be relatively narrow and peaks on forward and reverse strand should be separated by a region that corresponds approximately to the nucleosome bound DNA. This region is expected to be depleted of sequence reads (Figure 1). However, the distance between adjacent nucleosomes is usually short (~30 - 60 bp) and this may lead to overlap between peaks. When analysed at low resolution this can lead to the detection of extended enriched regions rather than individual nucleosomes.

In a related type of experiment a subset of nucleosomes is targeted through chromatin immunoprecipitation (ChIP) followed by high-throughput sequencing of ChIP fragments [5,6]. This technique, commonly known as ChIP-seq, has also been used to investigate the binding of transcription factors [7,8]. Due to the relatively short binding site of most transcription factors these studies typically produce wider, relatively isolated peaks in read counts compared to those obtained from nucleosome sequencing.

The increasing number of ChIP-seq and nucleosome positioning studies has led to the development of various approaches to analyse these data. The analysis typically starts by mapping short sequence reads to a reference genome, ignoring reads with non-unique alignments. To identify protein binding sites from these mapped reads, many commonly used methods generate a strand-independent profile of windowed read counts by recording the total number of reads on both strands that fall into a sliding window, or alternatively the number of overlapping extended reads for each position in the genome [5,7-11]. In both cases the height of the read count profile corresponds to the total number of reads in the window, irrespective of strand. The read extension method implicitly uses separate windows for the two strands, combining the sequence reads in both windows into a single read count. Explicitly using two distinct windows to construct the read count profile allows for the inclusion of information about the length of the protein binding site [4]. Peaks in the resulting read count profile are usually assessed for significance based on total read counts compared to a control sample [7,9], background model [8,10] or permutation of observed read counts [5]. The use of hidden Markov models (HMMs) [5,11] and kernel density estimators [12,13] has been suggested as an alternative way to identify significant peaks in the read count profile. Although this strand-independent approach to ChIP-seq analysis has been used frequently with apparent success, it ignores the fact that DNA binding proteins are expected to generate a similar number of reads on both strands adjacent to the binding site.

The need for a new approach to ChIP-seq analysis that incorporates the characteristics of protein binding sites has been recognised and has led to the development of new strand-specific methods. Kharchenko *et al*. [14] suggest three different approaches to utilise strand-specific read counts that outperform established strand-independent methods [7,8]. The SISSRs algorithm of Jothi *et al*. [15] considers the difference of read counts on both strands in a sliding window and locates potential protein binding sites by identifying sign changes in this net read count. Although these methods were developed with transcription factor analysis in mind, the methods presented in [14] are general enough to be extended to a nucleosome related analysis. The SISSRs algorithm assumes that binding sites are isolated, i.e., peaks from neighbouring binding sites do not overlap. This is unlikely to be true for nucleosomes since they are expected to be located close to each other, which restricts SISSRs to the detection of extended regions of enrichment. This makes SISSRs unsuitable for the identification of individual nucleosome positions. A similar issue arises with the method proposed by Zang *et al*. [16]. Although their method was specifically designed to identify histone modifications from ChIP-seq data it focuses on the detection of broad regions of enrichment rather than individual nucleosome positions. Spyrou *et al*. [17] propose a strand specific HMM analysis of ChIP-seq experiments and demonstrate its utility on transcription factor and histone modification data. Although Spyrou *et al*. make a deliberate effort to obtain results at high resolution the HMM framework limits the resolution that can be achieved by this method, which may be insufficient to distinguish between adjacent nucleosomes.

When sequencing nucleosomal DNA, either to determine nucleosome positioning or to identify specific histone modifications, it is important to realise that the binding site is substantially longer than for a typical transcription factor. While it may be acceptable to ignore the expected gap between peaks on forward and reverse strands when the binding site is small compared to the window used, as is often the case for transcription factor binding sites, this is not necessarily true for nucleosomes. Some studies choose the size of the sliding window to be the average DNA fragment length [5,8,9] while others choose a smaller window of 100 bp [7,10]. It should be noted that these window sizes are substantially larger than the transcription factor binding sites in question, but similar in length to or shorter than nucleosome-bound DNA. The use of 1 kb windows [5,9,11] or even 1 Mb windows [9] has been suggested. Although the increased window size alleviates the need to model the length of binding sites explicitly, it also leads to a notable decrease in resolution.

To locate nucleosomes at high resolution we propose to use an explicit model of protein binding site characteristics and of the resulting read patterns. This requires the model to be adjusted to the details of the experiment under consideration. In particular the impact of changes to the protocol used to isolate and purify nucleosomes has to be considered carefully. We demonstrate our approach to the high resolution analysis of ChIP-seq experiments by introducing ChIPseqR, an algorithm designed for the identification of nucleosomes. This method provides a number of parameters that allow it to be adjusted to different types of experiments but here we focus on the analysis of end-sequenced mononucleosomes after digestion with MNase. ChIPseqR is applied to simulated data on which it is shown to identify nucleosomes at high resolution while achieving better sensitivity and specificity than alternative methods. The favourable performance of ChIPseqR is confirmed through the application to end-sequenced mononucleosomes.

## Results

### Algorithm

#### Background distribution of sequence reads

To reliably identify nucleosome positions it is necessary to model the read counts associated with a binding event as well as read counts in the absence of protein binding. A frequently used assumption is that background sequence reads, which are unrelated to binding events, are independently and uniformly distributed throughout the genome [4,8,15]. This implies that the number of these background reads *X*_bg _starting at each position of the genome follows a Poisson distribution with constant rate parameter *λ*. Examination of the read density in negative control samples suggests a heterogeneous distribution of background reads [14]. To allow for this heterogeneity the use of a background model that incorporates changes in read density by assuming that λ follows a gamma distribution has been suggested by Ji *et al*. [10].

Here we propose a different approach to address this problem. Instead of estimating the background read density for the entire genome at once we estimate the local background read density in a window of width 2*w*_bg _+ 1. Consider *Y*_bg_(*i*) ~ Poisson(Λ_bg_(*i*)), the number of reads starting at position *i*. Then the number of background reads in a window centred at position *i *is

(1) $$X_{\text{bg}}(i)=\sum_{j=i-w_{\text{bg}}}^{i+w_{\text{bg}}}Y_{\text{bg}}(j)\sim \;\text{Poisson}\;(\lambda_{\text{bg}}(i))$$

with $\lambda_{\text{bg}}(i)=\sum_{j=-w_{\text{bg}}}^{w_{\text{bg}}}\Lambda_{\text{bg}}(i+j).$ For each position *i *the strand specific local background rate can be estimated as ${\hat{\lambda }}_{\text{bg}}(i)=x_{\text{bg}}(i),$ where *x*_bg_(*i*) is the observed number of sequence reads in window *i *on the relevant strand. This background model adapts to locally observed read densities without imposing a distribution on *λ*.

Confirming the observations made by Kharchenko *et al*. [14] we find that the background read density includes large peaks that are seemingly unrelated to the presence of nucleosomes. While the background estimation procedure described above adapts to changes in the background rate, the presence of large isolated peaks will lead to overestimation of the read rate for the surrounding area. A robust estimate of the background read rate is obtained by limiting the change in read rates between adjacent, non-overlapping, windows. If ${\hat{\lambda }}_{\text{bg}}(i-2w_{\text{bg}}-1)$ is positive we choose the robust estimate ${\tilde{\lambda }}_{\text{bg}}(i)$ as the largest value *j *such that $j\leq {\hat{\lambda }}_{\text{bg}}(i)$ and $P[X_{\text{bg}}(i)\leq j|\lambda_{\text{bg}}(i)={\hat{\lambda }}_{\text{bg}}(i-2w_{\text{bg}}-1)]\leq \gamma ,$ for an appropriately chosen probability λ.

#### Nucleosome model

A nucleosome will produce several DNA fragments in the sample. During the sequencing process short reads are produced from the 5'-ends of both strands of the DNA fragment population. The start position of sequence reads generated from this population corresponds to the start and end positions of DNA fragments in the reference genome. Although these may vary due to differences in fragment length and relative position of the nucleosome within the fragment, all read start sites will be located in proximity to the nucleosome but not within the strech of histone bound DNA itself. This leads to a region of increased read density on the forward strand upstream of the nucleosome which is mirrored by a region of increased read density on the reverse strand downstream of the nucleosome. The nucleosome, located between the two peaks in read density, is relatively depleted of sequence reads. This creates a distinctive pattern that can be partitioned into three regions: forward support (fwd), binding (bind) and reverse support (rev) region (Figure 1). Note that the forward and reverse support regions only cover the respective strand, allowing for overlapping peaks from neighbouring binding sites.

Consider a binding site of length *b *= 2*w*_bind _+ 1 starting at position *i *containing *X*_bind_(*i*) sequence reads from both strands, a support region on the forward strand of length *s *= 2*w*_sup _+ 1 starting at position *i *- *s *containing *X*_fwd_(*i*) forward strand reads and a support region on the reverse strand of the same length starting at position *i *+ *b *containing *X*_rev_(*i*) reverse strand reads. Here we assume that the read counts in these regions follow Poisson distributions, i.e., *X*_region_(*i*) ~ Poisson(λ_region_(*i*)), where *region *is one of *fwd*, *bind *or *rev*. Using the same assumptions as for the background distribution we consider *X*_region_(*i*) to be the sum of *w*_region _random variables *Y*_region _(*j*) ~ Poisson(Λ_region _(*i*)).

#### Scoring potential nucleosome positions

If a nucleosome is starting at position *i *the following three relations should hold: λ_fwd_(*i*) > λ_bg_(*i*), λ_bind_(*i*) < λ_bg_(*i*), λ_rev_(*i*) > λ_bg_(*i*). We assess these relations for each potential nucleosome position based on a likelihood ratio statistic, *W*_region_. This statistic is computed for each of the three regions of the potential nucleosome position, comparing the estimated read rate in the region to the estimated local background rate (see Methods). If a nucleosome starts at position *i *one would expect λ_fwd_(*i*) and λ_rev_(*i*) to be equal. To account for this and to avoid inflated values of *W*_fwd _in cases where a peak in read density in the forward support region is not matched by a comparable peak in the reverse support region, we use the truncated estimate

(2) $${\tilde{\lambda }}_{\text{fwd}}=\max\{j:0<j\leq {\hat{\lambda }}_{\text{fwd}}\;\text{and}\;P[X_{\text{fwd}}\leq j|\lambda_{\text{fwd}}={\hat{\lambda }}_{\text{rev}}]\leq \gamma \}$$

for a suitably chosen γ to calculate *W*_fwd_. The truncated estimate ${\tilde{\lambda }}_{\text{rev}}$ is defined similarly.

For each of the three regions a score *S*_region _is calculated. For the two support regions the score *S*_sup_, sup ∈ {fwd, rev}, is defined as

(3) $$S_{\sup}=\{\begin{array}{rr} +\sqrt{W_{\sup}}, & \text{if}\;{\tilde{\lambda }}_{\sup}>{\tilde{\lambda }}_{\text{bg}}\\ -\sqrt{W_{\sup}}, & \text{otherwise} \end{array}$$

and for the binding region as

(4) $$S_{\text{bind}}=\{\begin{array}{rr} -\sqrt{W_{\text{bind}}}, & \text{if }{\hat{\lambda }}_{\text{bind}}\;>{\tilde{\lambda }}_{\text{bg}}\\ +\sqrt{W_{\text{bind}}}, & \text{otherwise} \end{array}$$

The three region scores for each potential nucleosome are combined into a single nucleosome score

(5) $$S(i)=S_{\text{fwd}}(i)+S_{\text{bind}}(i)+S_{\text{rev}}(i).$$

Note that this score is only meaningful if there is at least one read in each of the two support regions and ${\tilde{\lambda }}_{\text{bg}}>0$.

#### Significance test

To assess the significance of nucleosome scores the distribution of *S *under the null hypothesis that no nucleosome starts at position *i *has to be determined. The nucleosome score for each position in the genome is the sum of the scores for the three regions of the potential nucleosome position (Equation (5)) and the score for each region is derived from the likelihood ratio statistic *W*_region _(Equations (3) and (4)). If the null hypothesis is true $W_{\text{region}}\dot{\sim }\chi_{1}^{2}\;\text{and}\;\text{thus}\;S_{\text{region}}\dot{\sim }\text{N}(\text{O},\;1).$ This might suggest the use of $S∼\text{N}(\text{0},\;\sqrt{3})$ to model the distribution of binding site scores under the null hypothesis. However, it is important to realise that, even under the null hypothesis, *S*_fwd _and *S*_rev _are unlikely to be independent due to the use of truncated rate parameter estimates, and the asymptotic results may not hold. To avoid misleading *p*-values that may result from the use of an incorrectly specified null distribution we generalize the above model to $S\sim \text{N}(\bar{\mu },\;\sigma ),$ where $\bar{\mu }$ is the sample median of the nucleosome scores and σ is estimated from the observed nucleosome scores. To obtain a good fit for the null distribution, and thus reliable *p*-values, it is necessary to identify a subset of scores that is representative of the null hypothesis.

Observing that the presence of nucleosomes will lead to larger nucleosome scores we truncate *S *at the τ^th ^and 50^th ^percentile, where 0 ≤ τ < 50 is chosen to exclude outliers in the lower tail while retaining a sufficiently large number of observations. This ensures that the vast majority of scores relating to protein binding events have no influence on the parameter estimation while also guarding against extreme values in the lower tail, which are observed when calculating nucleosome scores for positions that place unusually large numbers of sequence reads in the binding region. A maximum likelihood estimate for σ is obtained by fitting a truncated half-normal distribution to the selected subset of nucleosome scores.

The *p*-values for all observed nucleosome scores are calculated based on the estimated null distribution and corrected for multiple testing. To control the false discovery rate (FDR) we use a Benjamini-Hochberg procedure [18]. If the data contain many nucleosomes it may be beneficial to modify this procedure to account for the estimated proportion of true null hypotheses. This is done using the αAFDR approach discussed in [19], where it is shown to be equivalent to the procedure proposed by Storey *et al*. [20].

### Testing

To assess the performance of our method we compared it to a number of other peak-finding methods on simulated data. The three methods presented in [14], mirror strand peaks (MSP), mirror tag correlation (MTC) and window tag density (WTD), were chosen for comparison because they allow for strand specific peaks in read counts and employ different strategies to handle the expected gap between peaks. Briefly, WTD calculates a binding score based on the number of forward and reverse strand tags within a window upstream and downstream of a potential binding site. While this utilises the fact that a protein binding event should produce a peak in forward strand read counts upstream and a peak in reverse strand read counts downstream of the binding site, WTD does not require the two peaks to be similar in magnitude nor is the gap between peaks considered. MTC is similar to WTD but uses the correlation between forward strand and reverse strand reads to introduce a measure of similarity between the two peaks. Sequence reads close to the centre of the binding site are ignored when calculating the correlation coefficient which effectively introduces a gap between the two peaks. A Gaussian smoothing kernel is used by MSP to estimate local read densities for both strands and binding sites are required to lie between peaks of comparable magnitude. The R implementation of these methods provides the option to estimate the window size from the cross-correlation between the two strands. We found that this does not produce satisfactory results for the nucleosome data under consideration and used a 160 bp window instead. Unlike the methods proposed by Kharchenko *et al*. GeneTrack [13] was specifically designed for the detection of nucleosome positions. Like MSP, GeneTrack uses a Gaussian kernel to smooth read counts. The algorithm then proceeds to score each peak based on its height and reports the highest scoring set of non-overlapping peaks. GeneTrack makes no attempt to assess the significance of peaks, which results in a high number of nucleosome predictions but is likely to lead to many false positives if the data contains regions that are depleted of nucleosomes. To fairly evaluate GeneTrack's performance relative to methods that control the false discovery rate we choose a cut-off that leads to a similar number of significant predictions as the one produced by ChIPseqR. A further peculiarity of GeneTrack is that nucleosome predictions are centred on the peak in read counts. This results in predictions that are shifted by approximately half a nucleosome length compared to the actual position of the nucleosome. This inaccuracy was corrected for the purpose of this comparison. ChIPseqR was used with a binding region of 128 bp, support regions of 17 bp and a background window with *w *= 1000. These settings were found to perform well on real data (see Methods) and were used here for consistency.

All methods were applied to simulated data (see Methods) and various performance metrics are reported. The number of predicted nucleosomes at each level of coverage (Table 1) suggests very low sensitivity for WTD and MTC. At the lower coverage levels these methods failed to detect any nucleosomes and even for the 10 million read samples predicted nucleosome numbers remained very low. Better sensitivity was achieved by MSP and ChIPseqR. The lack of a significance threshold for GeneTrack makes it difficult to assess sensitivity in a meaningful way. However, a comparison of the percentage of stable nucleosomes identified suggests that GeneTrack's sensitivity is comparable with the one achieved by MSP at lower coverage levels and that it lies between the sensitivity of ChIPseqR and MSP for the 10 million read samples.

**Table 1:** Number of nucleosomes identified on simulated data by different methods at three levels of coverage

		ChIPseqR	GeneTrack	MSP	MTC	WTD
3 M reads	Sample 1	8,386	8,386*	4,809	0	0
	Sample 2	8,299	8,299*	4,726	0	4
	Sample 3	8,388	8,388*	4,892	0	0
	Total	25,073	25,073*	14,427	0	4
	% stable	24.9%	4.7%	5.4%	-	25%
	repeatability	0.07	0.02	0.007	-	-
6 M reads	Sample 1	6,257	6,257*	7,968	0	0
	Sample 2	6,286	6,286*	7,912	0	0
	Sample 3	6,431	6,431*	8,139	0	0
	Total	18,974	18,974*	24,019	0	0
	% stable	52.7%	6.7%	6.5%	-	-
	repeatability	0.16	0.05	0.014	-	-
10 M reads	Sample 1	19,907	19,907*	9,989	542	142
	Sample 2	20,359	20,359*	9,951	621	143
	Sample 3	20,223	20,223*	9,851	545	192
	Total	60,489	60,489*	29,791	1,708	477
	% stable	32.5%	18.2%	7.7%	68.1%	48.8%
	repeatability	0.31	0.16	0.06	0.10	0.04

*Cut-off was chosen to give the same number of significant predictions as ChIPseqR.

For each sample the number of predicted nucleosomes is reported. The total number of significant predictions for each level of coverage is reported together with the proportion of stable nucleosomes that were identified in all three samples as well as a measure of repeatability of stable nucleosome predictions between samples (see text for details).

**Figure 2:**
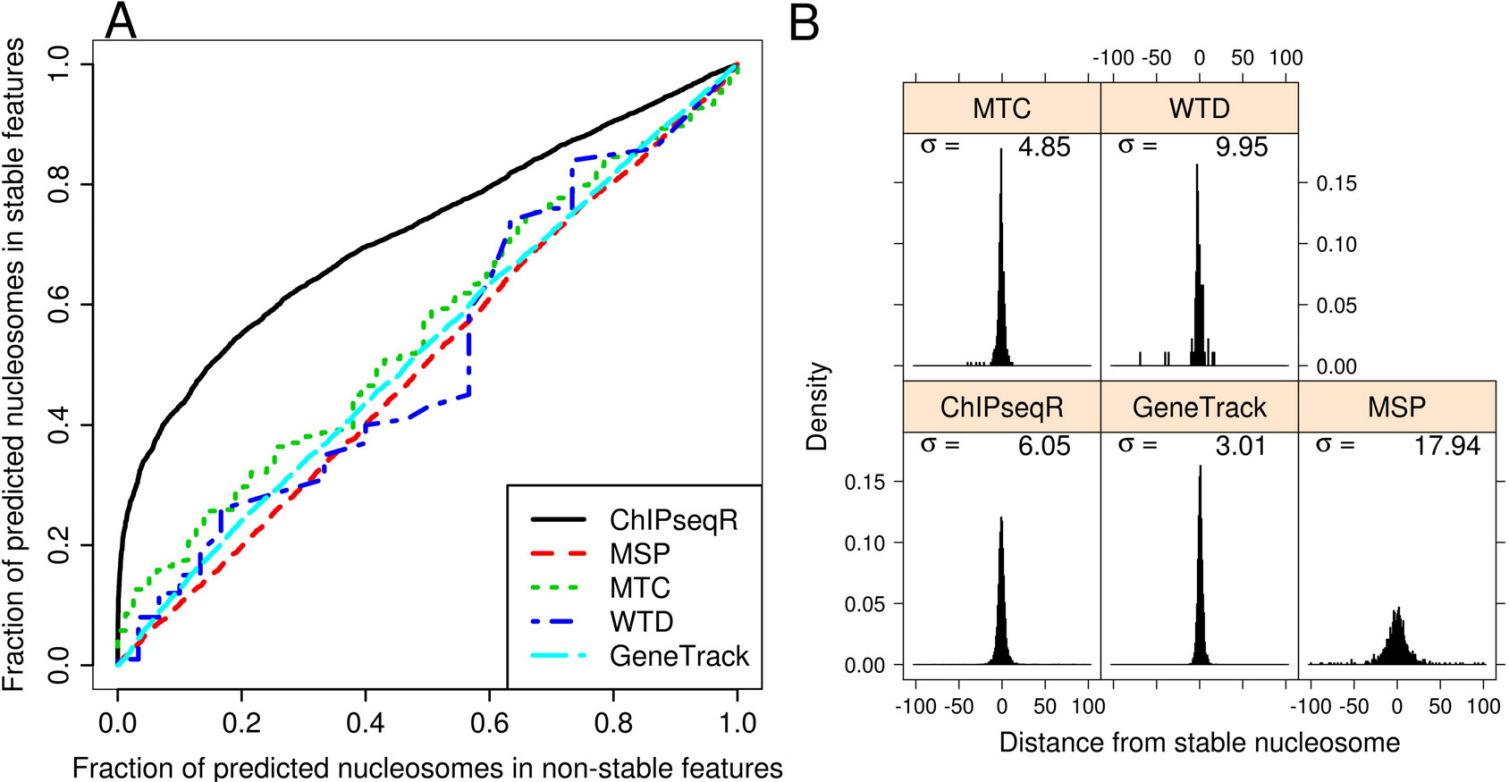
**Specificity and spatial resolution of nucleosome predictions**. (A) Fraction of predicted stable and non-stable nucleosomes considered significant at varying p-value cut-offs. A method that consistently produces smaller p-values for stable than for non-stable nucleosomes will result in a curve that gets closer to the top left corner than a method for which p-values and the stability of nucleosomes are unrelated. (B) Distance between nucleosome predictions from the third 10 M read sample and the location of stable nucleosomes. For each method observed distances are reported together with standard deviation of distances. A larger standard deviation corresponds to a lower resolution.

It may be instructive to consider the number of stable nucleosomes detected by each of the methods. For this purpose we considered a stable nucleosome to be detected if a given method produced a nucleosome prediction within 100 bp of the nucleosome centre. Although nucleosome predictions not corresponding to stable nucleosomes are not necessarily less valid, it is desirable to detect stable nucleosomes because their stability is likely to be related to a regulatory mechanism. Since the positions of these nucleosomes are stable it is straightforward to determine the distance between the location of the stable and predicted nucleosomes. MSP, GeneTrack and ChIPseqR produced a relatively large number of predictions at all coverage levels and identified increasing numbers of stable nucleosomes with increasing coverage. However, ChIPseqR consistently produced more stable nucleosome predictions than any of the other methods, indicating that it provides the best sensitivity of the methods in this comparison (Table 1). Assessing the specificity of the nucleosome predictions is less straightforward since many nucleosomes do not have well defined positions and therefore predictions cannot be classified as incorrect in the usual sense. Instead we again focused on the methods' ability to identify stable nucleosomes. A method that accurately locates nucleosomes rather than random positions along the genome is expected to predict the location of stable nucleosomes with high confidence as these should be relatively easy to identify due to the well defined peaks associated with them. As the stability of nucleosomes decreases the corresponding reads are increasingly dispersed and peaks become less obvious. To assess how well the different methods are able to separate stable nucleosomes from other nucleosomes we considered all nucleosomes predicted from the third 10 M read sample by each method and rank predictions by score. Figure 2 shows the fraction of predicted stable and non-stable nucleosomes considered significant with varying threshold. From this it is apparent that ChIPseqR not only identifies more nucleosomes than the other methods, it also is more reliable in terms of locating stable nucleosomes. Although GeneTrack produces a large number of nucleosome predictions, stable nucleosomes are not well distinguished from other nucleosomes.

To further investigate how reliably different methods identify stable nucleosomes we consider the stable nucleosomes identified from different samples at the same level of coverage (Table 1). For each of the three samples the stable nucleosomes identified by a given method are recorded such that *X_i_*(*j*) = 1 when the *j*^th ^stable nucleosome was detected in sample *i *and 0 otherwise. We assess a method's ability to reliably identify the same stable nucleosomes from different samples by computing the total correlation *C*_tot_(*X*_1_, *X*_2_, *X*_3_) [21] between the three samples. This provides a measure of the information shared between the samples, i.e. it will be maximised if the same set of nucleosomes is identified for each sample. To obtain a measure of repeatability that is comparable between methods and different levels of coverage regardless of the number of identified nucleosomes, we divide *C*_tot_(*X*_1_, *X*_2_, *X*_3_) by *C*_max_(*X*_1_, *X*_2_, *X*_3_) which is calculated as the total correlation of a permutation of *X*_1_, *X*_2_, *X*_3 _that maximises the number of repeated nucleosome detections. This normalises the repeatability such that it lies in [01]. Over 50% of the stable nucleosomes predicted by ChIPseqR from the 10 M read samples were successfully identified in all three samples which corresponds to a repeatability of 0.31. None of the other methods achieved a repeatability of more than 0.16. It is worth noting that while MSP produces a relatively large number of predictions they tend to differ substantially between samples, leading to a very low repeatability.

Another important performance measure is the resolution at which predictions are made, i.e., how close predicted nucleosome positions are to actual nucleosome locations. Peak-finding algorithms like the ones considered here typically involve a smoothing step to elucidate peaks. This comes at the cost of reducing the resolution of the raw data. To measure the resolution achieved by the four methods in this comparison we examined the distance between predicted stable nucleosomes and the underlying stable nucleosome positions (Figure 2). The best resolution was achieved by GeneTrack followed by MTC and ChIPseqR. While WTD appeared to produce predictions at an acceptable resolution, the low sensitivity of this method made an accurate assessment of the resolution difficult. The resolution of MSP was clearly the lowest in this comparison, limiting its usefulness for localising individual nucleosomes.

#### Characteristics of predicted nucleosome sites

To further investigate the utility of the proposed method we applied it to end sequenced mononucleosomes from *Arabidopsis thaliana*. Sequence reads were generated through Solexa sequencing of DNA fragments. Mononucleosome-sized DNA fragments were selected for sequencing after MNase digestion. Approximately 8 million uniquely mapped reads were used for the analysis.

**Figure 3:**
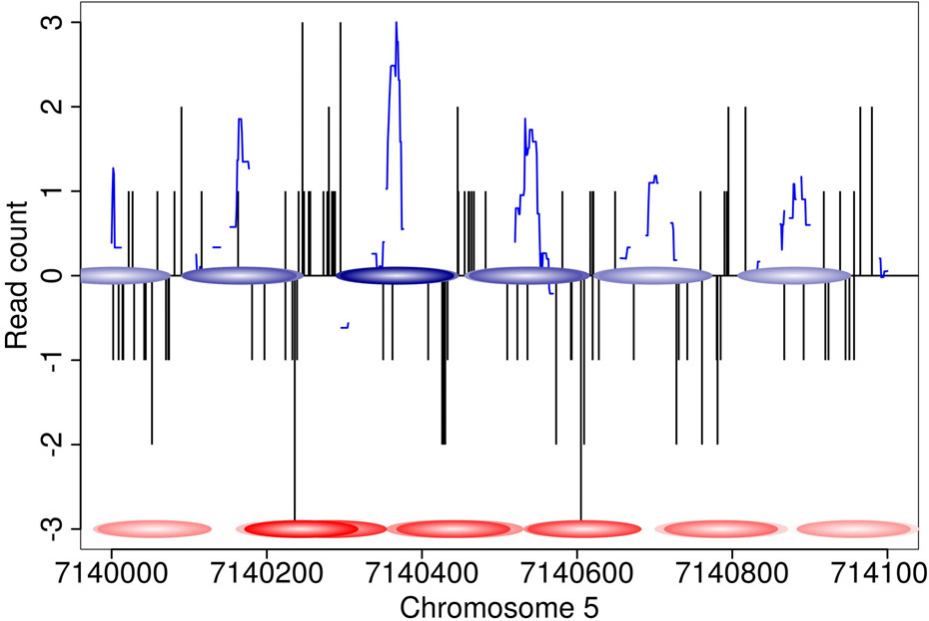
**Predicted nucleosomes**. Example of predicted nucleosomes from *Arabidopsis thaliana *dataset with *b *= 128 and *s *= 17. Vertical bars indicate read positions on forward (top) and reverse strand (bottom). The nucleosome score computed by ChIPseqR is shown as blue line. Blue ellipses indicate nucleosomes predicted by ChIPseqR. GeneTrack predictions are shown as red ellipses for comparison. The nucleosome shown in dark blue corresponds to a high confidence prediction (FDR < 0.05). Lighter shades of blue indicate lower confidence predictions.

The nucleosome model and scoring procedure described above require the specification of several parameters. We conducted computational experiments to investigate the influence of these parameters on the performance of ChIPseqR (see Methods) and chose a background window of 2001 bp, i.e. *w *= 1000 as well as two separate sets of parameters (*b *= 147, *s *= 10 and *b *= 128, *s *= 17) for the length of the binding and support regions to investigate the properties of nucleosomes predicted from end-sequenced mononucleosome fragments. The first set of parameters only produced about 2,200 significant nucleosome predictions while the second set identified 8,393 nucleosomes. An example of nucleosome predictions in a selected region of the genome is shown in Figure 3. Characteristics of interest are the distance between predicted stable nucleosomes, the location of stable nucleosomes relative to annotated genes and the presence of DNA sequence motifs within nucleosome sequences.

**Figure 4:**
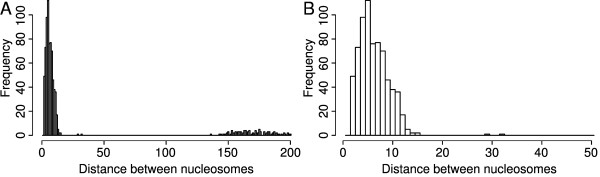
**Distances between adjacent nucleosome predictions**. Distribution of distances between adjacent nucleosome predictions for *b *= 128 and *s *= 17. Many adjacent nucleosome predictions correspond to overlapping nucleosomes, most likely due to variations in nucleosome position between cells. The shift in position between overlapping nucleosomes tends to be small with only ten cases where the shift is larger than 15 bp. Distances larger than 200 bp are not shown.

The distribution of distances between adjacent predictions should provide some insights into the distribution of stable nucleosomes throughout the genome. Note that, while individual nucleosomes along the same chromosome cannot overlap within a single cell, the position of nucleosomes is expected to vary between cells or even between different copies of a chromosome within the same cell to some degree. This may lead to overlapping nucleosome predictions if there is sufficient evidence to support these alternative positions. Figure 4 shows the distribution of distances between adjacent nucleosome predictions for *b *= 128 and *s *= 17. This distribution is characterised by a relatively large number of distances that are shorter than 15 bp followed by only ten cases with distances between 16 and 150 bp. The majority of distances between nucleosomes are larger than 200 bp. This suggests that some adjacent predictions correspond to adjacent stable nucleosomes while others have larger gaps between them. The relatively high abundance of overlapping predictions and the consistently small difference in position between them may represent some uncertainty about the location of these nucleosomes but also suggests that positioned nucleosomes are more likely to exhibit small rather than large changes in position depending on cell state. This observation is consistent with the findings of Albert *et al*. [1] regarding the translational and rotational settings of nucleosomes.

**Figure 5:**
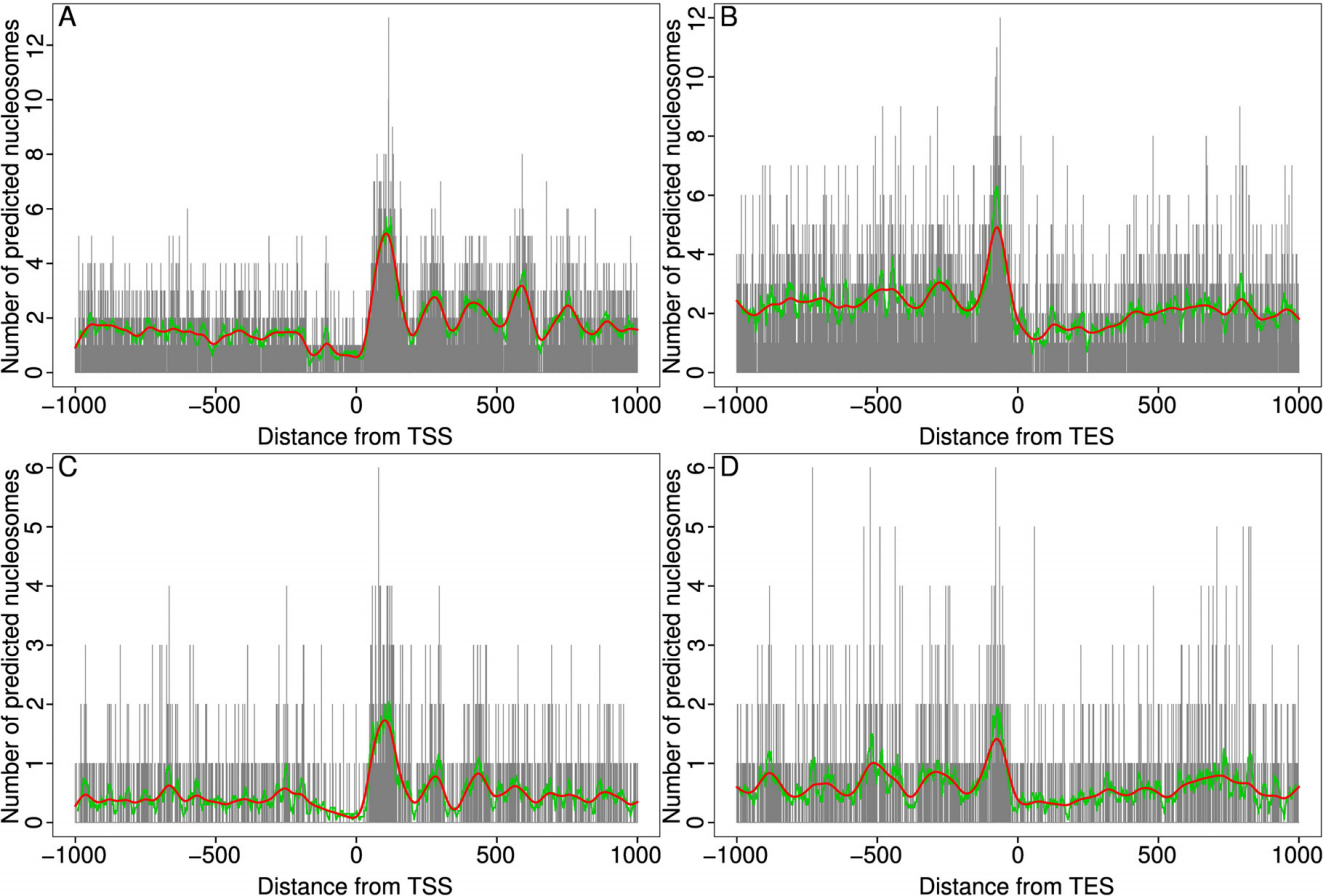
**Phasing of predicted nucleosomes**. Predicted nucleosomes are phased at transcription start and end sites. Predicted nucleosomes under both parameter sets ((*b *= 128, *s *= 17) (top) and (*b *= 147, *s *= 10) (bottom)) show clear evidence of phasing at TSS and TES. Vertical bars indicate the number of nucleosomes centred at each position. Red and green lines are smoothed nucleosome counts using smoothing splines and a moving average respectively.

Previous studies of nucleosomes in various organisms have found evidence for nucleosome positioning relative to transcription start sites [1-4]. To investigate whether there is evidence of positioning relative to the 5' and 3' end of annotated genes in the two sets of predicted nucleosomes, we aligned the transcription start sites (TSS) and transcription end sites (TES) of all annotated *Arabidopsis *genes and determined the number of predicted nucleosomes centred at each position within 1 kb of the respective feature (Figure 5). There is clear evidence for positioned nucleosomes at both ends of genes. The +1 nucleosome is followed by a series of positioned nucleosomes of decreasing stability. The region upstream of the +1 nucleosome is depleted of nucleosomes. There is some evidence for nucleosome positioning upstream of the nucleosome free region (NFR) although this is much weaker than for the +1 nucleosome. The nucleosome positions predicted with *b *= 128 show some evidence of a nucleosome located in the NFR similar to the -1 nucleosome reported for some genes in humans [4].

It has been suggested based on theoretical considerations as well as empirical findings that stable nucleosomes are associated with a periodic pattern of dinucleotides [1,2,22]. To assess whether similar patterns play a role in nucleosome positioning in *Arabidopsis *and whether they can be detected based on the stable nucleosomes predicted by our method, we used the over 2200 non-overlapping nucleosome positions identified with *b *= 147, *s *= 10 to investigate the properties of nucleosome sequences. To this end the position specific frequency of all dinucleotides was determined in a ±500 bp window around the centre of predicted nucleosomes (see Methods).

**Figure 6:**
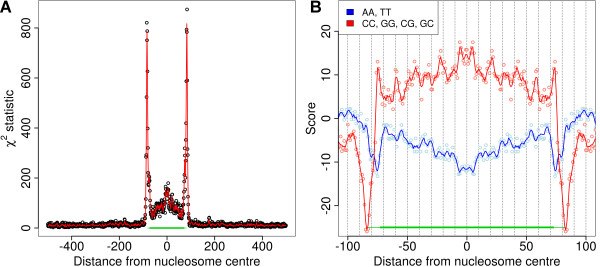
**Dinucleotide frequencies in nucleosome sequences**. (A) DNA sequences associated with predicted stable nucleosomes show clear evidence of specific dinucleotide patterns. (B) AA/TT and CC/GG/CG/GC dinucleotides show a periodic pattern in nucleosome sequences. In both panels open circles correspond to data points while solid curves show a three base pair moving average.

Dinucleotide frequencies throughout the predicted nucleosome sequences differ markedly from genome-wide frequencies while there is little evidence for this outside the nucleosome region (Figure 6(A)). Note that the A/T bias of MNase leads to a notable increase in the frequency of related dinucleotides in the flanking regions of predicted nucleosomes. Studies in yeast and *Drosophila *have reported a periodic pattern of A/T and C/G dinucleotides in nucleosome sequences [1,2]. We observe a similar pattern that is dominated by alternating AA/TT and CC/GG dinucleotides (Figure 6(B)). These dinucleotides correspond to a subset of previously reported nucleosome-associated patterns. This may be an indication that such patterns vary between species as suggested by Kogan and Trifonov [23]. They also find AA to be the dominant pattern associated with periodic dinucleotide signals at gene splice sites in *Arabidopsis*. The periodic patterns observed in nucleosome binding DNA are accompanied by an increase in G/C content compared to the genome average (Figure 6(B)), which is consistent with previous observations in yeast [24,25] and humans [26].

#### Verification of predicted nucleosome positions

Nine genomic regions containing 13 nucleosome predictions with different levels of confidence were selected for qPCR verification. Out of the 13 predicted nucleosomes 11 were verified (see Methods).

The DNA abundance profiles obtained through qPCR show several well defined peaks. Many of these correspond to predicted nucleosomes while others appear to suggest the presence of adjacent nucleosomes that were not predicted by the initial analysis. To investigate this further we identified peaks in DNA abundance that may correspond to positions of additional nucleosomes that do not overlap predicted nucleosomes and tested them for significance using the same procedure as above. This results in the detection of additional nucleosomes in 10 of 14 potential nucleosome positions. The remaining four locations are characterised by flat DNA abundance profiles and may correspond either to nucleosome free regions or areas of delocalised "fuzzy" nucleosomes (see additional file 1: Figure S1).

### Implementation

An R package implementing the method described here is freely available from Bioconductor and from the authors' web page at http://www.bioinformatics.csiro.au/ChIPseqR together with simulated datasets.

## Discussion

Unlike the majority of existing methods for the analysis of ChIP-seq experiments the novel approach presented here is suitable for the detection of nucleosome positions. The explicit modelling of nucleosomes increases the sensitivity and makes it possible to detect their positions at low coverage levels. Combined with the relatively small amount of smoothing required for the analysis this enables ChIPseqR to reliably identify nucleosomes at high resolution. This is especially important for the analysis of nucleosome positions and histone modifications since this typically requires the sequencing of a much larger proportion of the genome than would be required for a transcription factor study, which results in lower coverage for the same amount of sequencing. Furthermore, nucleosome studies aim to distinguish binding sites that are usually located close to each other and may vary in location throughout the sample. It is therefore important to identify binding sites at high resolution and to allow for partially overlapping peaks in read counts associated with adjacent binding sites.

### High resolution and repeatability

Although all methods compared here use strand specific read counts and all except WTD are able to handle overlapping peaks from adjacent binding sites only ChIPseqR achieved good sensitivity and high resolution. The high resolution achieved by ChIPseqR is further highlighted by the detection of periodic dinucleotide patterns in the DNA sequence of predicted binding sites. Strong evidence of these patterns was found without any further sequence alignment, suggesting that the predicted positions of stable nucleosomes are highly accurate. This result is also indicative of high specificity since false positives would dilute any sequence motifs. It should be noted that all methods in the comparison would benefit from a further increase in coverage. We expect that differences in performance will diminish as coverage saturates but achieving sufficient coverage to reach saturation is still costly which makes it desirable to obtain reliable results from low coverage data.

Another performance measure of interest is an algorithm's ability to produce consistent results for repeated experiments. This is of particular interest when low coverage data is used since this is likely to result in the prediction of only a subset of all binding sites. Despite this one would like to obtain results that do not depend too much on the details of a particular sequencing run, i.e., the analysis should be robust towards moderate changes in read position. This is important to enable researchers to draw reliable conclusions from the analysis that can be expected to hold when the experiment is repeated. Although all methods in the comparison perform better than random, indicating that they all provide a core of consistently predicted nucleosomes, ChIPseqR provides substantially higher repeatability than any other method at all levels of coverage.

### Analysis of mononucleosomes

**Figure 7:**
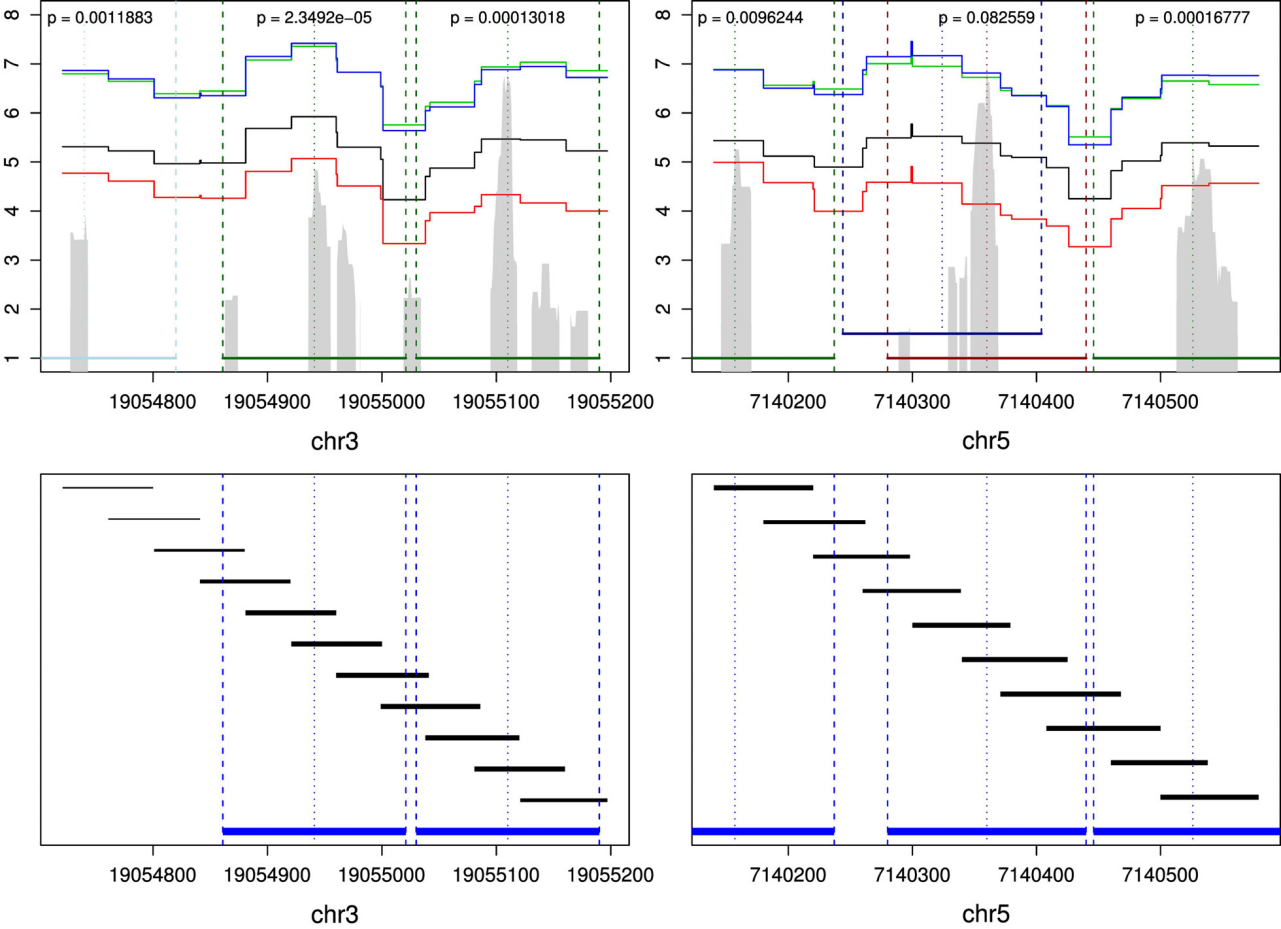
**Results of qPCR verification and amplicon design**. Two of the regions chosen for verification are shown with log mean quantities of DNA measured on four replicates. The location of verified nucleosomes is indicated by dark green horizontal bars. A nucleosome prediction that is not supported by qPCR measurements is shown in red with an alternative position suggested by the PCR results indicated in blue. A light blue bar indicates the location of a nucleosome identified by qPCR that was not predicted by our analysis. Nucleosome scores are shown shaded in grey. Below the location of amplicons used to tile across the selected regions is shown relative to predicted nucleosomes.

The favourable performance of ChIPseqR observed in the comparison on simulated data is confirmed by its application to the mononucleosome dataset. Predicted nucleosomes are found close to transcription start and transcription end sites that are known to be associated with stable and phased nucleosomes [1-4], suggesting that ChIPseqR is well suited for the detection of moderately well positioned nucleosomes. This is confirmed by the results of the qPCR verification of selected nucleosome predictions. Considering the verification results it is evident that predicted nucleosomes corresponding to pronounced peaks in qPCR measurements, which are likely to represent stable or phased nucleosomes, were confirmed reliably. Closer inspection of the two unverified nucleosome predictions reveals that one of them is located within 30 bp of a significant peak in DNA abundance (Figure 7 and additional file 1: Figure S1). It seems likely that the location of this nucleosome would be predicted more accurately with increased coverage. It should be noted that the peak in qPCR measurements appears to be wider than expected for a stable nucleosome and may suggest the presence of an alternative nucleosome position in the sample. The other unverified nucleosome position is located at what appears to be the start of a "fuzzy" nucleosome region. This makes it difficult to assess the exact location with any method. We note that the analysis of the short read data produced three distinct peaks in the binding site score located close to the predicted binding site (see additional file 1: Figure S1). This is consistent with the presence of overlapping nucleosome positions that one would expect from delocalised nucleosomes.

The regions chosen for qPCR verification were selected to encompass predictions with a range of FDRs. The fact that all low confidence predictions were verified suggests good specificity even for relatively high values of the nominal FDR. Consequently the FDR cut-off chosen for the initial analysis may be considered conservative.

### Influence of model parameters

When discussing the performance of ChIPseqR it is important to realise that this depends on a number of parameters. Apart from the coverage provided by a given dataset, some of the model parameters will impact on the performance characteristics. Choosing *b*, the length of the binding region, close to the actual binding site length will result in high resolution and is expected to improve specificity. However, this will reduce the method's ability to detect less stable binding sites, thus reducing sensitivity. Such unstable binding sites can be detected more reliably if *b *is reduced while the length of the support regions is increased. This increases sensitivity but may lead to reduced resolution. It should be noted that the choice of *b *= 128 that was determined to be optimal for the data considered here is less than the 147 bp that may be expected for a nucleosome. However, this reduced binding region is consistent with the presence of alternative preferred positions of a stable nucleosome located ±10 bp from a central position that have been observed previously [27].

Another important factor is the estimation of the background read rate λ_bg_. There are at least two different approaches to the estimation of this parameter. The one taken here relies on choosing a large window relative to the binding site which is applied to the same sample that contains the binding sites. The relatively large size of the background window results in an average read rate close to a base level even if there are binding sites within the window. However, the presence of binding sites will increase the estimated background rate above the actual background, thus producing conservative binding site scores. An alternative approach would use a separate control sample to assess the background read rate. This has the potential to produce more accurate results and is generally recommended but we note that it may be difficult and costly to obtain good control samples for this purpose. Even when a suitable control sample is available the issue of an appropriate normalisation method for this type of data has not been settled (see [28] for a discussion of some problems with current approaches). We therefore consider the single sample method presented here to be more practical at the moment, especially for nucleosome sequencing.

## Conclusions

The model proposed here was specifically designed for the analysis of end sequenced nucleosomes after MNase digestion. Although several ChIP-seq analysis algorithms are available for tasks like this they are typically designed for the analysis of transcription factor binding. Our analysis demonstrates that it is beneficial to take the substantial differences between transcription factor binding and nucleosome positioning experiments into account explicitly. Although some existing methods may be general enough to handle both transcription factor ChIP-seq and nucleosome sequencing data they will be outperformed by more specialised methods, especially on low coverage data. Although GeneTrack was designed for the analysis of nucleosome experiments and has been used for this purpose with apparent success [1] it does not take advantage of the characteristics of nucleosome experiments. This results in a performance similar to the general ChIP-seq methods.

The main advantages of the model presented here are the high resolution of nucleosome predictions and its ability to operate on low coverage data. This makes it especially suitable for the analysis of nucleosome positions in situations where it is difficult to obtain high coverage, i.e. many reads per nucleosome position, because the genome under consideration is large or the sample contains a mixture of many cells (and therefore many different nucleosome configurations).

The most important parameters of our model, *b *and *s*, are closely related to the underlying biology and length of DNA fragments used for sequencing. This makes ChIPseqR a flexible and versatile method for the analysis of nucleosome positioning and makes it possible to adapt the method to a range of different experiments. We have demonstrated its favourable performance in the context of nucleosome positioning experiments and similar performance is expected for other nucleosome related experiments, such as histone modification studies. More generally it should be possible to apply the approach demonstrated here to other ChIP-seq experiments. We note however that the binding site model should be adjusted to accommodate the differences in experimental procedures, especially when DNA is fragmented through sonication rather than MNase digestion.

## Methods

### Simulated data

Data were simulated using the ChIPsim R package (available at http://www.bioinformatics.csiro.au/ChIPsim and from Bioconductor) with default parameters. Briefly, ChIPsim generates a sequence of nucleosome features to cover a given genome. Each type of feature has its own characteristics, corresponding to different chromatin structures commonly observed in nucleosome positioning experiments. Here we distinguish between 'stable', 'phased', 'fuzzy' and 'nucleosome free' regions. The simulation assumes that sequence reads are generated from a population of cells, i.e., nucleosome positions are represented by distributions rather than discrete locations.

For this study we generated nucleosome features to cover the *Arabidopsis *genome. These features include 13,758 stable nucleosomes, 16,313 regions of phased nucleosomes covering 82 Mb, 6,360 regions of fuzzy nucleosomes covering 32 Mb and 11,293 nucleosome free regions covering 1.3 Mb. A dataset containing 30 million sequence reads, each 36 bp long, was generated from the TAIR 8 assembly of the genome ftp://ftp.arabidopsis.org/Sequences/wholechromosomes/. From this smaller datasets with three, six and ten million reads were created with three replicates at each level of coverage. All simulated datasets are available online from http://www.bioinformatics.csiro.au/ChIPseqR.

### Mononucleosome data

To generate the mononucleosome fragments for end-sequencing and qPCR verification a lysate was prepared by grinding 1 g total seedling material of *Arabidopsis *ecotype Columbia to a fine powder in liquid nitrogen. The ground material was added to 10 ml lysis buffer (50 mM HEPES pH7.5, 150 mM NaCl, 1% (v/v) Triton TX-100, 0.1% (w/v) sodium deoxycholate, 0.1% (w/v) sodium dodecyl sulphate, 1× Plant Proteinase Inhibitor mix (Sigma St Louis, MO). 1 ml aliquots of the lysate were digested with 7.5 to 30 units micrococcal nuclease (Fermentas, Vilnius, Lithuania) for 15 minutes at 37°C, followed by proteinase K treatment and phenol extraction. DNA fragments were separated by agarose gel electrophoresis and the fragments in the size range of mononucleosome cores (~150 bp) were excised and purified. The purified mononucleosome cores were then used to generate a library for Illumina GS sequencing (carried out by Geneworks, Adelaide, Australia) or used for qPCR quantification. The data from Illumina GS sequencing are archived at the NCBI Sequence Read Archive (SRA) under accession number SRP001458.

### Verification of predicted nucleosome positions

Nine genomic regions containing a subset of predicted nucleosomes were selected for qPCR verification. Regions were chosen to cover predictions with FDR values between 0 and 0.2 and large enough to contain between ten and twelve tiled amplicons. The resulting regions are between 438 bp and 563 bp in length and contain a total of 13 nucleosome predictions. Of these predictions four have p-values between 0 and 0.01, five have p-values between 0.01 and 0.05 and the remaining four predictions have p-values between 0.05 and 0.2.

A PCR-based strategy was used to verify the nucleosome positions predicted from the analysis of the sequence data. A set of predicted nucleosome positions were selected and short (~80 bp) PCR amplicons were designed across each position with each amplicon shifted by ~40 bp relative to the previous one. The amplicons were designed so that two amplicons should fall within the boundaries of each predicted nucleosome position. A listing of primer sequences and positions of amplicons are shown in additional file 2: Table S1, the locations of predicted nucleosomes covered by amplicons are given in additional file 3: Table S2. The position of amplicons relative to predicted nucleosomes in two regions are shown in Figure 7. A quantitative PCR assay was then used to determine DNA abundance in four replicate mononucleosome isolations compared to uncut genomic DNA standards. Regions that are contained in the mononucleosome fractions should show enrichment in this assay. Similar results were obtained when micrococcal nuclease-digested DNA was assayed without purification of the mononucleosome cores (data not shown). The mean abundance of DNA was measured for each amplicon in four replicates. These measurements were converted into a continuous abundance profile for each replicate and region by averaging the abundance of different amplicons where they overlap. The resulting profiles were log transformed to account for differences in scale between replicates (Figure 7). To determine whether a predicted nucleosome position was confirmed by the qPCR data the DNA abundance at the centre of the predicted nucleosome was compared to the abundance at positions ±80 bp from the centre, which correspond to predicted linker DNA. The difference in abundance was tested for deviation from 0 at a significance level of 0.01 using a one-sided paired *t*-test. Predictions associated with significantly higher DNA abundance at the centre of predicted nucleosome cores than in the corresponding linker region were considered to be verified. Following this procedure eleven of the 13 nucleosome predictions were verified (Table 2).

**Table 2:** Number of predicted and verified nucleosomes in three significance bands

	[0, 0.01)	[0.01, 0.05)	[0.05, 0.2)	total
predictions	4	5	4	13
verified	3	4	4	11

Predicted binding sites with corresponding p-values ranging from 0 to 0.2 representing high, medium and low confidence predictions were chosen for verification.

#### Quantitative PCR Assays

DNA protected from micrococcal nuclease digestion was quantified using an Applied Biosystems 7900HT realtime PCR machine. PCR conditions were 1× PCR buffer (Platinum Taq buffer, Invitrogen, CA), 3.5 mM MgCl_2 _0.2 mM dNTP mix, 0.4 *μ*M oligonucleotide primers, 1:20000 (v/v) SybrGreen, 0.025 U/*μ*l Platinum Taq. Nucleosome core samples were quantified against undigested genomic DNA standards.

### Choosing model parameters

The binding site model and scoring procedure described above require the specification of several parameters. In this section we discuss possible choices of parameter values and their impact on the analysis. The parameters of the model itself are the length of the binding site *b*, the length of the support region *s *and the length of the background window *w*. The method used to score potential binding sites introduces a number of additional parameters, namely γ_bg _and γ_sup _to specify the cut-off in difference between rates for the background and supporting region read rate estimates and the cut-off *τ *used to exclude outliers when fitting the null distribution.

To obtain a better understanding of how these parameters affect the analysis we considered a range of parameter combinations. The performance of different sets of parameter values was assessed based on the number of binding sites identified in the mononucleosome data and accompanying control sample where the latter are assumed to be false positives. We note that the coverage in the control sample is substantially lower than in the treatment. This may have an impact on the number of identified binding sites but we expect the general trends identified here to hold for control samples with higher coverage. In the following analysis we used a nominal false discovery rate of 0.05 and *τ *= 0.95. Since the length of nucleosome-bound DNA is known to be about 147 bp we initially chose *b *= 147 and *s *= 15. To investigate the effect of γ_bg _and γ_sup _we predicted binding sites using values of 0.5, 0.9, 0.99 and 1 for each of these parameters while the width of the background window was set to 1001 bp, 2001 bp and 3001 bp. The number of binding sites predicted in the treatment and control sample for all parameter combinations were recorded and compared to determine a set of parameters that provides high specificity and sensitivity. At this stage of the analysis we are mainly concerned with identifying parameter values that keep false positive predictions to a minimum while maintaining an acceptable number of predicted binding sites in the treatment sample, i.e. the focus is on specificity. To assess specificity we considered the ratio between the number of predicted binding sites in the control and treatment. Lower values of this ratio indicate higher specificity.

**Figure 8:**
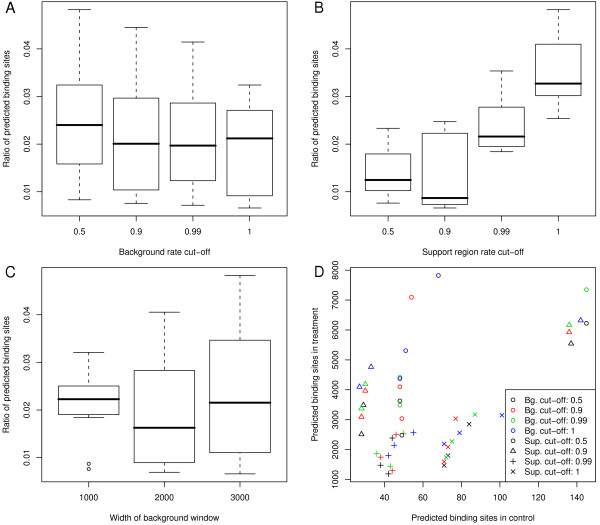
**Number of binding sites identified in treatment and control sample using different sets of parameters**. (A - C) Box-plots show the ratios of predicted binding site counts in treatment and control achieved with different parameter choices. Lower values suggest higher specificity. (D) The number of predicted nucleosomes in the control sample is plotted versus the number of nucleosomes identified from the mononucleosome sample. Different colours correspond to different background rate cut-offs while different symbols indicate different cut-off levels for support region rates.

Results are summarised in Figure 8. The choice of γ_sup _had the largest effect on specificity with γ_sup _= 0.9 generally outperforming the alternatives. The impact of other parameters was less pronounced. A background window width of 2001 bp appeared to perform slightly better than 1001 bp and 3001 bp windows. The choice of γ_bg _had very little influence on the specificity. The results suggest a small increase in specificity for γ_bg _= 0.99 and γ_bg _= 0.9 We note that an increasing value of γ_bg _tends to increase the number of predicted binding sites while also increasing the influence of unusually large peaks in the data. This may become more apparent as the overall coverage increases. Based on these considerations we chose γ_bg _= 0.9. as the conservative alternative.

**Figure 9:**
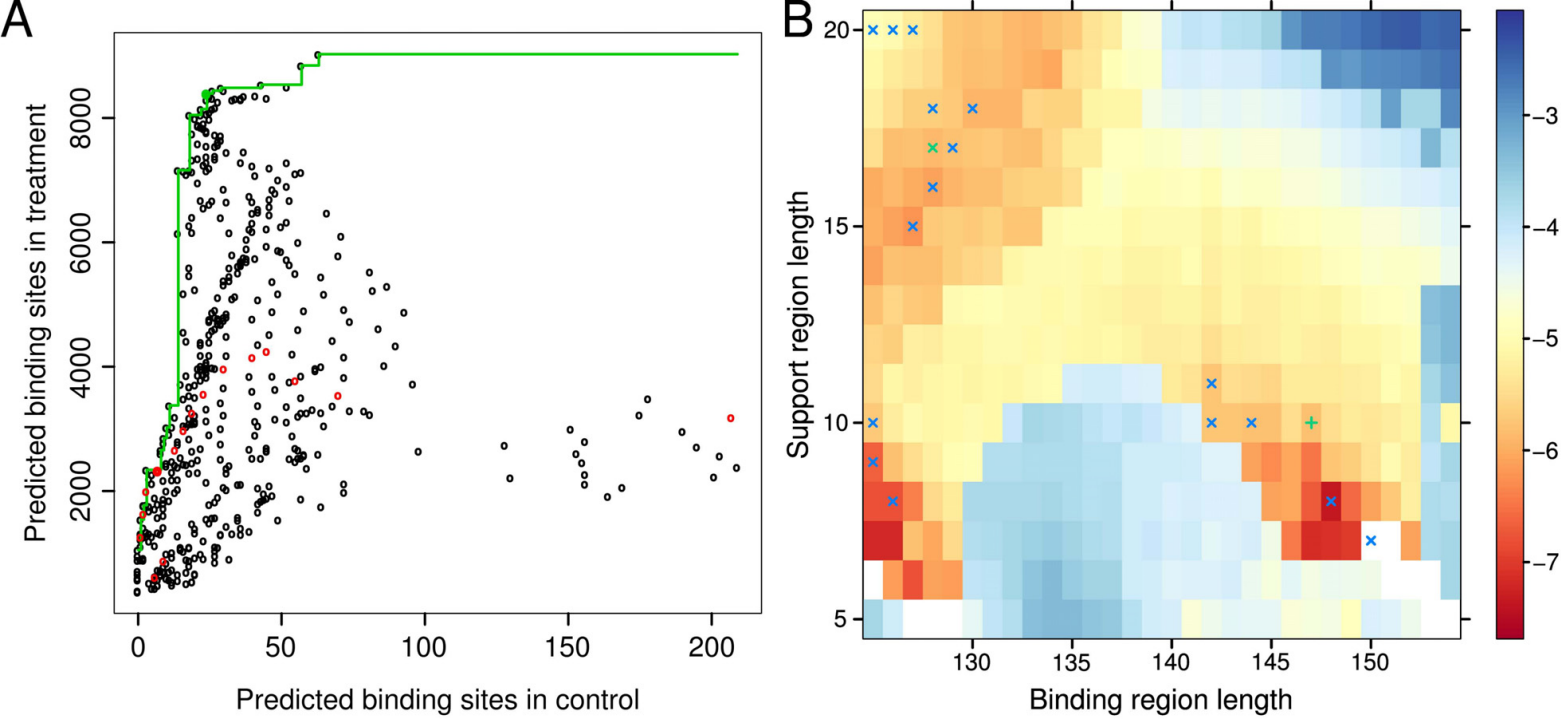
**Number of nucleosomes identified in treatment and control with varying binding and support regions**. (A) The scatter plot shows all combinations of nucleosome predictions in treatment and control obtained for the chosen range of parameter values. Points corresponding to maximum number of predicted nucleosomes in the treatment sample for a given number of predicted nucleosomes in the control are marked in green. Results from parameter sets where *b *= 147 are highlighted in red. Solid points indicate parameter combinations that were selected for further analysis. (B) Log ratio of the number of nucleosomes identified in treatment and control for each combination of binding and support region length. Parameter combinations corresponding to green points on the left are marked with ×. Green symbols indicate parameter combinations chosen for further analysis.

Using the parameter values determined above we then considered different values for the length of binding and support regions. While the length of DNA bound as part of a nucleosome is well established, it may be beneficial to use a shorter binding region. The sample under consideration contains a mixture of cells from various tissue types. With the position of most nucleosomes expected to vary between tissue types, or even between cells of the same type, only the most stable nucleosomes will produce a sequence read pattern with a binding region of 147 bp. As the stability of a given nucleosome decreases the resulting pattern will show a smaller binding region and larger support regions. We investigated how combinations of *b *∈ {125, . . . , 154} and *s *∈ {5, . . . , 20} affect the number of nucleosomes detected in treatment and control. For each combination of *s *and *b *the number of predicted nucleosomes in treatment (**m**) and control (**M**) was recorded, aiming to determine values for *b *and *s *that provide a favourable trade-off between sensitivity and specificity. Consider (*M_i_*, *m_i_*), the pair of nucleosome counts produced by the *i*^th ^parameter set. For each *M_i _*∈ **M **we determined *m_k _*such that *m_k _*is the largest value in **m **for which *M_k _*≤ *M_i_*. Then

(6) $$ℱ=\{(M_{k(i)},\;m_{k(i)})\;:\;k(i)=\arg\max[m_{j}|1\leq j\leq 480\;\text{and}\;M_{j}\leq M_{i}],\;1\leq i\leq 480\}$$

is a set of points representing parameter combinations that are optimal in the sense that neither sensitivity nor specificity can be improved without adversely affecting the other performance measure. While all points in ℱ are optimal in the above sense, some combinations of parameter values will be more useful in practice than others. Using *b *= 128 and *s *= 17 provides a favourable trade-off between the number of nucleosomes identified in treatment and control (Figure 9). We note that none of the parameter sets with *b *= 147 is in ℱ; indeed over 70% of optimal parameter combination have 125 ≤ *b *≤ 130. This suggests that only few nucleosome positions are maintained throughout the sample. Nucleosome positions predicted in the treatment sample with *b *= 128, *s *= 17 and *b *= 147, *s *= 10 were selected for further analysis.

### Scoring potential nucleosomes

To obtain the statistic for the comparison of read counts in two regions, consider the independent random variables *Y*_0*j *_~ Poisson(Λ_0*j*_) and *Y*_1*j *_~ Poisson(Λ_1*j*_). We then obtain two independent random variables *X*_0 _and *X*_1 _by summing over *j*:

(7) $$X_{k}=\sum_{j=1}^{t_{k}}Y_{kj}\sim \;\text{Poisson}\;(\lambda_{k}),$$

where $\lambda_{k}=\sum_{j=1}^{t_{k}}\Lambda_{kj}=t_{k}{\bar{\Lambda }}_{k}$ with ${\bar{\Lambda }}_{k}$ the average of the Λ*_kj _s*, *t_k _*= 2*w_k _*+ 1 and *k *= 0, 1. If we let $\phi =\frac{{\bar{\Lambda }}_{1}}{{\bar{\Lambda }}_{0}}=\frac{t_{0}\lambda_{1}}{t_{1}\lambda_{0}}$ then the probability of observing *X*_0 _= *x*_0 _and *X*_1 _= *x*_1 _is given by

(8) $$P\left[X_{0}=x_{0},X_{1}=x_{1}|\phi ,\lambda_{0}\right]={\left(\frac{t_{1}}{t_{0}}\right)}^{x_{1}}\frac{\lambda_{0}^{x_{0}+x_{1}}\phi^{x_{1}}}{x_{0}!x_{1}!}e^{-\lambda_{0}}e^{-\phi \lambda 0\frac{t_{1}}{t_{0}}}$$

Thus, the log likelihood is

(9) $$\ell_{1}\left(\phi ,\;\lambda_{0}\right)=-\lambda_{0}+\left(x_{0}+x_{1}\right)\ln\lambda_{0}-\phi \lambda_{0}\frac{t_{1}}{t_{0}}+x_{1}\ln\phi +x_{1}\ln\frac{t_{1}}{t_{0}}-\ln[x_{0}!x_{1}!]$$

from which maximum likelihood estimates for *ϕ *and λ_0 _are obtained as

(10) $$\hat{\phi }=\frac{x_{1}t_{0}}{x_{0}t_{1}}$$

(11) $${\hat{\lambda }}_{0}=x_{0}$$

If ${\bar{\Lambda }}_{0}$ and ${\bar{\Lambda }}_{1}$ are known to be equal, i.e. *ϕ *= 1, we obtain

(12) $$\ell_{0}(1,\;\lambda )=-\lambda +\left(x_{0}+x_{1}\right)\ln\lambda -\lambda \frac{t_{1}}{t_{0}}+x_{1}\ln\frac{t_{1}}{t_{0}}-\ln[x_{0}!x_{1}!]$$

and

(13) $$\hat{\lambda }=t_{0}\frac{x_{0}+x_{1}}{t_{0}+t_{1}}$$

The likelihood ratio statistic can then be written as

(14) $$\begin{array}{c} W=\;2\;\{\ell_{1}(\hat{\phi },{\hat{\lambda }}_{0})-\ell_{0}(1,\hat{\lambda })\}\\ =2\{x_{0}(\ln x_{0}-\ln t_{0})+x_{1}(\ln x_{1}-\ln t_{1})\\ +(x_{0}+x_{1})(\ln[t_{0}+t_{1}]-\ln[x_{0}+x_{1}])\} \end{array}$$

If ${\bar{\Lambda }}_{0}$ and ${\bar{\Lambda }}_{1}$ are indeed equal, the asymptotic distribution of *W *is known to be $\chi_{1}^{2}.$

### Scoring of nucleosome sequence motifs

The observed frequencies of dinucleotides in DNA that is predicted to be part of the nucleosome core are compared to the genome-wide frequencies by calculating a position specific dinucleotide score *S_ij_* (Equation (15)).

(15) $$S_{ij}=\frac{o_{ij}-e_{j}}{\sqrt{e_{j}}},$$

where *o_ij _*is the number of times dinucleotide *j *is observed at position *i *and *e_j _*is the number of times one would expect to see dinucleotide *i *at any given position based on its relative abundance in the genome. Then the statistic *S_i _*(Equation (16)) is asymptotically *X*^2 ^and can be used to determine regions where dinucleotide frequencies differ significantly from the background.

(16) $$S_{i}=\sum_{j}S_{ij}^{2}\dot{\sim }\chi_{15}^{2}$$

## Authors' contributions

PH developed and implemented the model and carried out the analysis. CAH conceived, designed and carried out the experiment. PH and CAH wrote the manuscript. DB and GS provided supervision to PH. All authors approved the final manuscript.

## Supplementary Material

Additional file 1 **Results of qPCR verification**. The regions chosen for verification are shown with log mean quantities of DNA measured on four replicates. The location of verified nucleosomes is indicated by dark green horizontal bars. Two nucleosome predictions that are not supported by qPCR measurements are shown in red with an alternative position suggested by the PCR results indicated in blue for the nucleosome in region 6. Light blue bars indicate the location of nucleosomes identified by qPCR that were not predicted by our analysis. Click here for file

Additional file 2 **Primer sequence and location for amplicons used for qPCR verification of selected regions**. Click here for file

Additional file 3 **Location and p-value of predicted nucleosomes selected for verification**. Click here for file
