# Supplementary figures and images for: ChIPseqR: analysis of ChIP-seq experiments
Source: BMC Bioinformatics. 2011 Jan 31;12:39. doi: 10.1186/1471-2105-12-39 (PMC3045301; doi:10.1186/1471-2105-12-39)

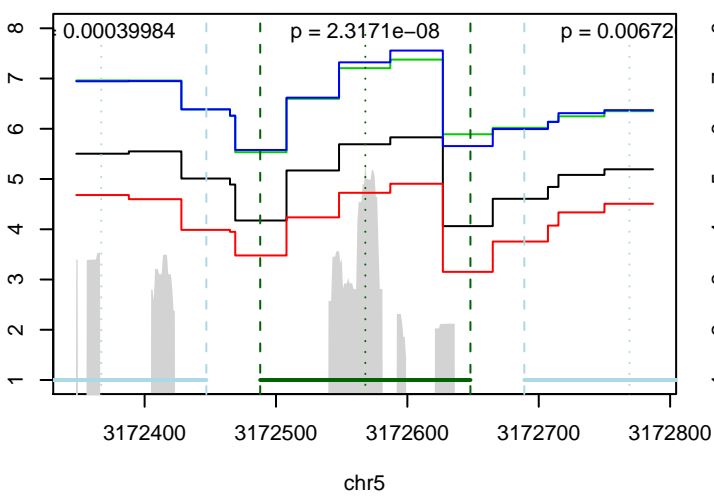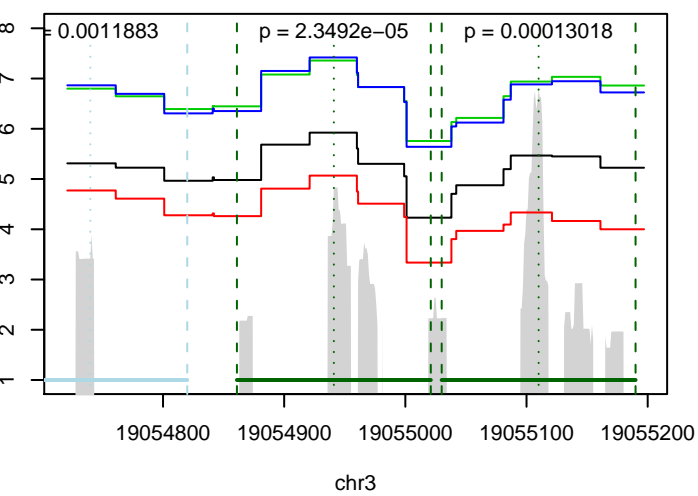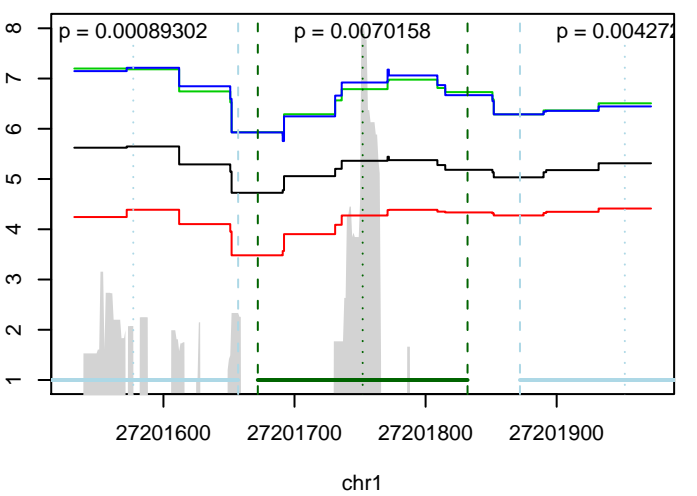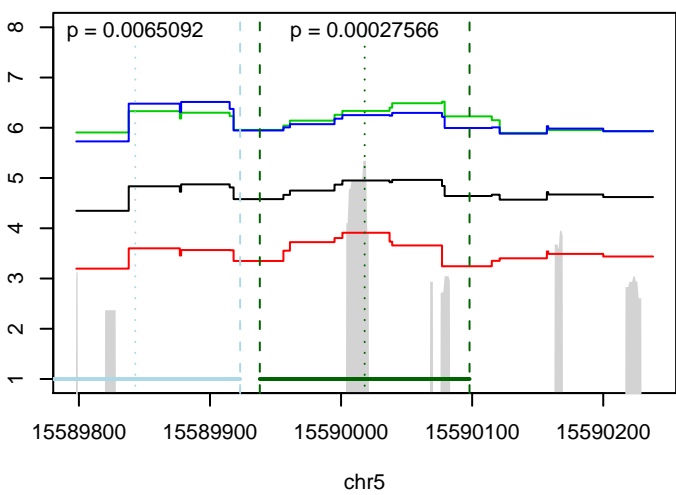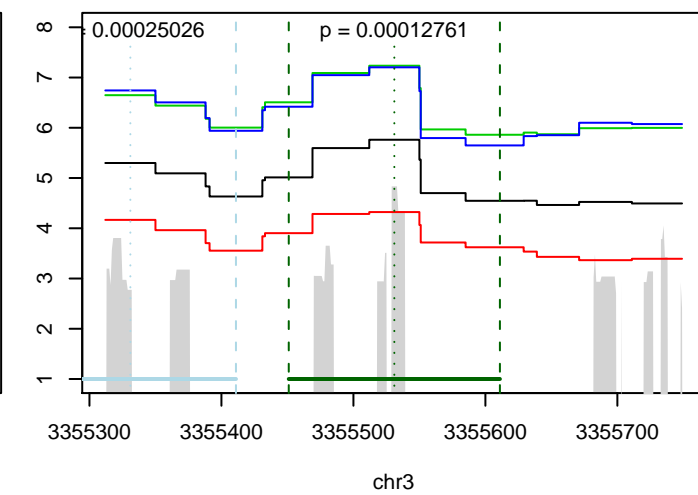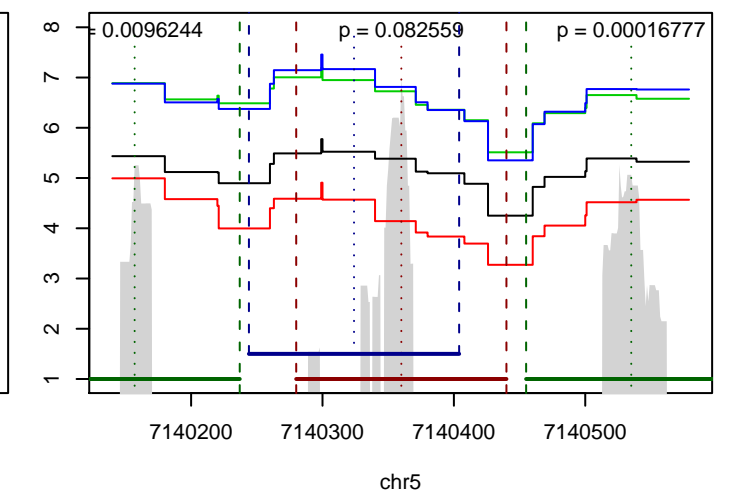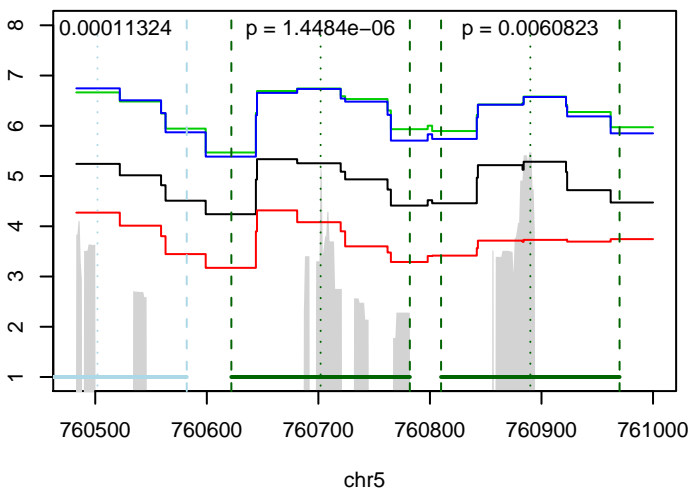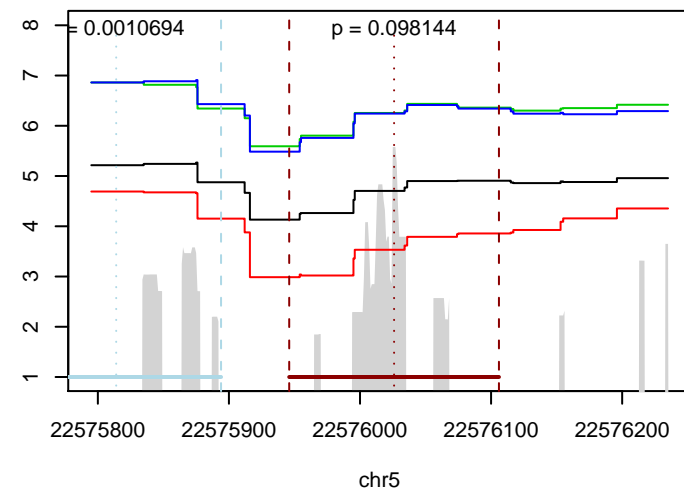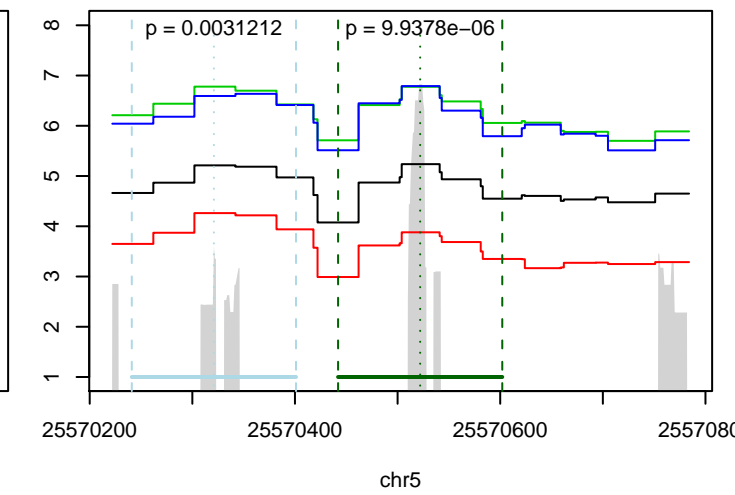

Supplement: Additional file 1 — Results of qPCR verification. The regions chosen for verification are shown with log mean quantities of DNA measured on four replicates. The location of verified nucleosomes is indicated by dark green horizontal bars. Two nucleosome predictions that are not supported by qPCR measurements are shown in red with an alternative position suggested by the PCR results indicated in blue for the nucleosome in region 6. Light blue bars indicate the location of nucleosomes identified by qPCR that were not predicted by our analysis. [file 1471-2105-12-39-S1.PDF]
